# Supplementary material for: Are carbon emissions trading and green financial instruments synergistic? -Comprehensive quantitative research based on content analysis
Source: PLoS One. 2024 Mar 7;19(3):e0298601. doi: 10.1371/journal.pone.0298601 (PMC10919601; doi:10.1371/journal.pone.0298601)
Supplement: S1 Data — (ZIP) [file pone.0298601.s001.zip › Data/地级市Z_F工作报告.zip!/║■▒▒/╬Σ║║╩╨/└┤╫╘╣1⁄2╓┌║┼í╛┬φ┐╦╩2╛▌═°í┐_2017.docx]

各位代表：

现在，我代表市人民政府，向大会报告工作，请予审议，并请市政协各位委员和列席人员提出意见。

一、2016年和过去五年工作回顾侯卫东官场笔记

过去的一年，我们认真贯彻落实习近平总书记系列重要讲话精神和治国理政新理念新思想新战略，在省委省政府和市委的坚强领导下，积极应对经济下行和特大洪涝灾害，勇于创新、克难奋进，实现了“十三五”良好开局。地区生产总值11912亿元，增长7.8%。

——发展新动能加快形成。高新技术产业产值8446亿元，占规模以上工业总产值比重达到64%，高新技术产业、现代服务业等新动能占经济总量比重达到60%。

——供给侧结构性改革成效明显。“三去一降一补”扎实推进。钢铁行业提前完成去产能任务。企业直接融资规模1459.9亿元，增长11.3%。打好“营改增”、降低涉企收费等“组合拳”，为企业减负175亿元。

——对外开放实现新突破。以武汉片区为主体的中国（湖北）自由贸易试验区、武汉新港空港综合保税区获批，武汉成为内陆唯一拥有两个综合保税区的城市。口岸出入境人数251.7万人次，居中部城市第一。

——抗洪救灾取得重大胜利。去年，我市遭遇历史罕见的特大洪涝灾害，一度周降雨量592.3毫米，为武汉有气象记录以来最高值。全市上下众志成城，打赢了邾城保卫战，确保了20万群众生命财产安全；打赢了天河机场保卫战，确保了国际国内航班正常运行；及时处置了倒口湖管涌群等236处各类险情，确保了城市安全。及时转移安置群众，紧急救助灾民25万人次，倒损房屋群众全部搬入新居。军民并肩奋战，共同谱写了新世纪抗洪壮歌！

——市民生活品质不断提高。轨道交通6号线、机场线通车试运营，地铁日均客运量207万乘次，增长25.8%，市民出行更加舒适便捷。中山大道完成改造，百年老街焕发新颜，东湖绿道建成开放，深受市民欢迎。新、改、扩建公园15个,新增绿地783万平方米，市民更好亲近自然。精准脱贫年度任务超额完成，减少贫困人口45739人、贫困村77个。

一年来，我们主要抓了以下工作：

一是迎难而上稳增长促转型。突出抓好“6+6+8”等一批重大增长点和重点企业，突出抓好服务业升级，经济运行缓中趋稳、稳中向好。

重大产业项目建设实现突破。华星光电六代线、东风雷诺等5个投资50亿元以上工业项目建成投产，为近年最多。新签约引进7个投资50亿元以上、6个投资30—50亿元先进制造业项目。存储器项目开工建设，总投资240亿美元，为新中国成立以来我市最大的单体投资项目。

主要支柱产业发展实现突破。汽车及零部件产值突破3000亿元，汽车产量176.7万辆，信息技术制造产值突破2000亿元。金融机构本外币存贷款余额首次双超2万亿元，存款余额增速居副省级以上城市首位，贷款余额增速居第2位。社会消费品零售总额5610.6亿元，旅游业增加值、软件和信息服务业收入均超过千亿元。农产品加工业产值2682.1亿元。

二是先行先试抓创新强驱动。全面创新改革试验方案获国务院正式批复，“中国制造2025”城市试点示范、投贷联动试点等10项国家授权先行先试改革任务落地实施。

创新人才创投资本加速汇聚。认定“城市合伙人”116名，新引进创新创业领军人才251名，其中国家千人计划人才13名，留汉就业创业高校毕业生15万人，创历史新高。全市各类创投机构724家，管理资本规模过千亿元。

双创平台不断完善。东湖新技术开发区成为全国首批双创示范基地。启动3批共13个“创谷”建设。各类孵化器221家，总面积超过千万平方米，居全国城市首位。举办“黄鹤杯”高层次人才创新创业项目大赛等双创活动，新增大学生创业企业2100家。

企业创新主体作用日益增强。新增国内外500强和跨国公司研发机构30个、各类科技创新平台201个。发明专利申请量20643件，增长36.9%，企业申请量占一半以上。高新技术企业新增521家，累计2177家。涌现出超高速超大容量超长距离光传输、首台常温常压储氢·氢能汽车等一批世界领先的自主创新成果。

三是突出重点抓改革扩开放。统筹推进100项重点改革任务，着力提升开放功能，发展活力显著增强。

重点领域改革取得突破。推进区级行政审批3.0改革全覆盖，实现审批职责、事项、环节全集中；推进“双随机一公开”监管创新，80%的执法部门建立“一单两库”。改革市、区财政体制，深化专项资金管理。拓宽民间投资领域，启动10个PPP项目试点，民营众邦银行获准筹建。新增各类市场主体14.61万户，增长12.3%。社会治理“1+10”改革取得新进展。深化法治政府建设，办结人大议案2件、代表建议283件，政协建议案3件、提案615件，满意率均达到99%以上。

开放型经济加快发展。实际利用外资增长16.1%，引进世界500强企业13家，累计243家。开通5条国际航线，累计41条。中欧（武汉）国际货运班列业务延伸到亚欧28个国家。英国在汉设立总领事馆。新增国际友好城市、国际友好交流城市19个。武汉首届国际马拉松赛等重大国际性赛会成功举办。

四是城建跨越强功能提品质。保持建设势头，城建投资突破2000亿元。

一批重大城建交通项目建成投用。天河机场第二跑道投入使用，机场旅客吞吐量突破2000万人次。武孝城际铁路通车运营。阳逻港集装箱铁水联运项目开工建设。第三轮轨道交通建设规划的10条线路全部开工，轨道交通运营里程达到181公里。四环线西段主线贯通，汉阳大道改造、东风大道高架二期等项目竣工。

市政保障功能不断提升。新增排水干网105公里、污水管网107公里,在建排涝能力490立方米/秒，污水处理厂提标在建9个。加快智慧城市建设，在全国率先推出“易行江城”智慧交通、渣土车智能管控等智慧应用系统，拥堵延时指数全国城市排名继续下降。

生态环境品质明显改善。认真接受中央环保督察，全年清查整改环境突出问题92个。空气质量优良天数237天，比上年增加45天，优良率64.8%,比上年提高12.2个百分点，PM10、PM2.5平均浓度分别下降11.5%、18.6%，空气质量全国74个城市排名前移7位。水环境质量逐步好转，长江、汉江岸线资源环境整治取得明显成效。对牛山湖实施永久性退垸还湖。深化“绿满江城、花开三镇”行动，造林绿化4.1万亩，中心城区花卉面积增加8万平方米。

五是民生优先增福祉促和谐。新增就业17.9万人，扶持创业2.66万人。企业退休人员人均养老金、城乡居民基本养老保险基础养老金标准分别提高8%、25%。建成保障房5.5万套。新、改、扩建53所公益普惠性幼儿园，完成46所中小学标准化建设。公立医院综合改革全面推开，市、区属公立医院全面取消药品加成。武汉中共中央机关旧址纪念馆建成开放。“平安武汉”建设进一步深化，食品药品安全保障水平不断提升，安全生产形势保持平稳。第七届世界军人运动会场馆建设全面启动。再次荣获“全国双拥模范城”称号。

国家安全、国防动员、军民融合、民防、消防、仲裁、保密、气象工作积极推进，参事、文史、档案、民族、宗教、侨务、地方志工作得到加强，妇女、儿童、老龄、残疾人事业健康发展。

回望2016，矛盾风险挑战巨大，稳增长保平安考验严峻。全市上下团结一心、砥砺前行，焕发出锐意进取的昂扬斗志，展现出奋发有为的精神风貌，稳定了经济社会发展大局。在这里，让我们为各行各业的出色业绩点赞！

各位代表！2016年经济社会的健康发展，标志着本届政府工作圆满收官。本届政府任期的五年，是武汉发展史上不平凡的五年。我们在以习近平同志为核心的党中央坚强领导下，按照省委省政府要求和市委决策部署，紧紧依靠全市人民，敢为人先、追求卓越，奋力建设国家中心城市、复兴大武汉。武汉发展恢弘壮丽、阔步前行，经济实力不断增强，城市面貌日新月异，战略地位显著提升。

这五年，经济大发展。我们围绕产业转型升级，大力推进工业倍增、服务业升级计划。工业投资累计1.15万亿元，18个投资50亿元以上项目建成投产，千亿元产业增加到5个。金融、物流、工程设计等现代服务业加快发展。武汉经济总量迈上万亿台阶。

这五年，城市大蝶变。我们围绕提升功能，大力推进城建跨越。城建投资累计7846.9亿元。地铁由线成环、通达三镇，中心城区快速路网加快形成，水陆空国际大通道不断拓展，城市更新改造步伐加快，大都市风貌日渐彰显。

这五年，文明大提升。我们围绕提高城市品质，大力推进文明创建，成功跨入全国文明城市行列。深入开展城管革命，城乡环境面貌焕然一新，荣获“国家卫生城市”称号。物质文明精神文明实现双丰收。

这五年，民生大改善。我们围绕提升市民生活水平，大力发展社会事业。全市公共财政用于民生领域支出累计4122亿元，年均增长18.6%。就业社保和社会救助体系不断完善，教育、卫生、文化、体育等公共服务均衡优质发展，农村生产生活条件明显改善。荣获全国综治最高荣誉“长安杯”。

回首过去，每一个武汉人都是这段光荣历程的亲历者、见证者、推动者，我们无愧于这座伟大的城市！

各位代表！历尽天华成此景,人间万事出艰辛。这五年，武汉取得的每一项成就，都是广大干部群众奋力打拼的结果。在此，我代表市人民政府，向全市人民，向给予政府工作大力支持的人大代表、政协委员，向各民主党派、人民团体和各界人士，向中央在汉单位和各类驻汉机构，向驻汉人民解放军、武警官兵，向参与城市建设的劳动者，向所有关心支持武汉发展的港澳台同胞、海外侨胞和国际友人，表示崇高敬意和衷心感谢！

我们也清醒地认识到，发展中的困难和挑战不少。综合经济实力与标兵城市差距较大，传统产业转型升级艰难，部分行业受多种因素影响大幅下滑，部分主要经济指标没有实现预期；民营经济发展不充分，经济外向度、国际化水平亟待提升；精细化管理水平和城市品质有待提高，山水资源禀赋彰显不够，交通拥堵、城市渍水、环境污染等短板亟待补齐；农民增收后劲不足，城乡二元结构依然突出；教育、医疗、养老等公共服务与群众期待还有差距；少数干部担当拼搏精神不足，不想为、不会为、不敢为现象依然存在，隐形变异“四风”问题还时有出现。对此，我们将采取有效措施，切实加以解决。二、今后五年工作展望二号首长

各位代表！习近平总书记2013年视察湖北武汉时，多次对“复兴大武汉”给予肯定，并提出了“四个着力”的要求。未来五年，是落实新发展理念、加快复兴大武汉的关键时期。市第十三次党代会提出了今后五年武汉发展的战略目标：到2018年，地区生产总值和城乡居民收入比2010年翻一番以上，人民生活品质明显提升，脱贫攻坚任务全面完成；到2021年，高水平全面建成小康社会，加快建设现代化、国际化、生态化大武汉,为复兴大武汉打下坚实基础。

围绕这一战略目标，政府工作的总体要求是：高举中国特色社会主义伟大旗帜，深入贯彻习近平总书记系列重要讲话精神和治国理政新理念新思想新战略，统筹推进“五位一体”总体布局，协调推进“四个全面”战略布局，坚持稳中求进工作总基调，按照省委省政府建设“五个湖北”和“强化主中心”“充分发挥核心带动作用”的要求，在市委领导下，聚力改革创新，奋力拼搏赶超，加快建设国家中心城市，高水平全面建成小康社会，全面开启复兴大武汉新征程，让宏伟蓝图一步一步变为现实，使群众期盼变为美好生活。

为此，今后五年我们将主要推进八大任务落实。

第一，着力推进结构优化、产业升级，打造全国重要的经济中心。大力振兴实体经济。加快发展先进制造业，一手抓战略性新兴产业培育，一手抓传统产业改造升级，促进生产型制造业向服务型制造业转型，推动服务型制造业与生产性服务业深度融合、制造业与互联网物联网深度融合。做大做强汽车及零部件、信息技术制造、装备制造、生物医药等产业，形成2个产值超5000亿元和若干超2000亿元产业。加快建设具有全球影响力的产业创新中心，高新技术产业产值达到1.7万亿元。推进现代服务业高端化发展，努力建设全国重要的商贸物流中心、区域金融中心、世界设计之都。提升现代都市农业发展水平，建成国家现代农业示范区。

大力推动民营经济创新发展。实施“聚商育商工程”，以发展民营高科技企业为重点，集聚培育一批行业领军型、创新引领型民营企业，大力培育和引进中国民营企业500强，建设民营经济创新发展新高地。大幅降低民间投资准入门槛、大幅降低企业负担，大力支持民营企业做大做强，依法保护民营企业合法权益，不断提高民营经济比重和地位。

第二，着力推进创新驱动、人才支撑，打造全国重要的科技创新中心。建设一批国家制造业创新基地、大科学装置。支持武大、华科大等高校“双一流”建设。打造享誉世界的“光谷”，建设“中国车都”。大力推动招才引智。深化“城市合伙人”计划，引进10名产业科学家、1000名创新创业领军人才。打造15个“创谷”，加快建设大学生创业街区园区、环高校创新带。采取有力举措，五年吸引100万大学生在汉创新创业，把武汉打造成大学之城、青年之城、梦想之城、创新之城！

第三，着力推进先行先试、重点突破，争当全面深化改革“排头兵”。深化供给侧结构性改革，深入推进“三去一降一补”，健全要素市场，引导资源配置，提高供给质量。深化国资国企改革，努力培育全国布局、行业领先的企业集团。深化“放管服”改革，建立“网上审批为常态、网下审批为例外”的4.0审批服务新体系，打造审批项目最少、审批速度最快、服务质量最优的城市。努力在创新政府治理、建立完善生态补偿和考核机制、创新驱动发展体制机制、创新社会治理等16项改革上率先取得重大突破。

第四，着力推进双向开放、优势重塑，打造内陆开放高地。高标准建设自贸试验区，把武汉片区建设成为全国全省对外开放的试验田、新窗口。把招商引资作为赶超发展的“一号工程”，来汉投资世界500强企业超过300家。外贸总额、对外直接投资额实现翻番。拓展国际大通道大平台，国际客货运航线达到70条以上。争取更多领事机构、国际性组织和有影响力的国际性活动落户武汉，打造国际交往中心。努力将第七届世界军人运动会办成世界水平、中国特色、精彩纷呈、非凡卓越的国际体育盛会。过去大武汉主要大在开放上，历史上一度“驾乎津门，直逼沪上”。我们要更高水平引进来，更大步伐走出去，重构大武汉开放格局，从内陆城市再次走向开放前沿！

第五，着力推进空间优化、标杆引领，统筹提高城市发展水平。强化武汉在全国发展大局中的战略地位。全面提升创新引领、交通枢纽、产业带动、要素集散功能，深入推进武汉大都市区一体化发展。发挥武汉“主中心”作用，有力促进中部崛起；发挥武汉核心作用，有力挺起长江经济带“脊梁”。

优化城市空间格局。构建“1个主城+3个副城+3个新城组群”的全域城市体系。支持江岸、江汉、硚口、汉阳、武昌、青山、洪山区提升现代服务业规模水平，支持东西湖、蔡甸、江夏、黄陂、新洲区规划建设中等规模的现代化新城。加快推进“三旧”改造。

建设世界一流的城市亮点区块。按照“共抓大保护、不搞大开发”要求，规划建设长江新城，形成基础设施体系框架，形成支撑性高效产业雏形，努力打造代表城市发展最高成就的展示区、全球未来城市的样板区。规划建设武汉长江主轴，全面规划长江市域段，重点围绕主城区段，与交通轴线、城市阳台、防洪设施等建设相结合，建成国家5A级江汉朝宗文化旅游景区、汉口历史文化风貌街区，推进汉口滨江国际商务区、汉正街中央服务区、武昌滨江文化商务区建设取得重大进展，打造城市交通轴、发展轴、文化轴、生态轴、景观轴，努力建设世界级城市中轴文明景观带。规划建设东湖城市生态绿心，建成百里东湖绿道，完成景中村改造，建设国家级生态湿地公园和国际知名的生态旅游风景名胜区，努力打造世界级城中湖典范。

加快建设全国性综合交通枢纽。推进两纵两横两连“米”字形高铁网规划建设，打造全国高铁中心、全国性铁路路网中心。建设中部门户枢纽机场，天河机场旅客年吞吐能力达到4500万人次，积极推动高铁进机场。构建面向流域、对接国际的港口集群、航运网络和服务平台，基本建成长江中游航运中心。每年至少通车2条地铁线，实现“主城联网、新城连通”，运营里程超过400公里，加快打造世界级“地铁城市”。畅通二环、提升三环、建成四环、东扩外环，新建5条以上放射性道路，打造环射成网、循环连通的“五环十八射”快速路网体系。

第六，着力推进生态建设、环境保护，打造滨水生态绿城。深入实施“拥抱蓝天”“拥抱绿水青山”行动。空气质量优良率力争提高到80%。推进“四水共治”，治理水环境，保障水安全，做好水文章。新增污水管网570公里，污水处理厂尾水标准全面实现一级A排放；新增排水干网300公里，新增抽排能力1000立方米/秒；新、改、扩建供水管道170公里。构建多级生态绿地体系，新建绿道1300公里，打造天兴生态绿洲。我们要呵护好宝贵的山水资源，建设滨水生态绿城，把山水留在城市，让“武汉蓝、江城绿、百湖清”成为美丽中国的一道靓丽风景！

第七，着力推进文化繁荣、文明建设，全面提升文化软实力。大力弘扬社会主义核心价值观，深入实施公民道德建设工程，注重家庭、家教、家风，不断提升市民文明素质。加快建设以琴台中央文化艺术区为核心，以武昌古城—环东湖和沙湖、汉口沿江—张公堤和园博园为支撑的“一核两带”文化功能集聚区。实施“文化+”战略，深化文化与科技融合，扶持文艺精品创作，引导城乡居民扩大文化消费，推动文化产业成为支柱产业，打造全国重要的文化创意中心。

第八，着力推进富民惠民、共建共享，切实增强市民获得感幸福感。坚持以人民为中心的发展思想，制定实施解决好人民群众普遍关心的突出问题工作方案，多谋民生之利，多解民生之忧，让市民享有与城市跨越发展相对应的生活水平。实施城乡居民增收激励计划，提高就业和收入水平。养老保险全覆盖、医疗保险补短板、住房保障提水平，建立更加公平更可持续的社会保障制度。推动教育服务质量大幅跃升。推进惠民强医，推广健康管理，建设健康武汉。

各位代表！规划未来是为了创造未来。未来的武汉，一定会经济更发达、城市更美丽、生活更舒适、社会更公平。一个现代化、国际化、生态化的大武汉，正向我们走来！三、2017年重点工作安排

2017年是新一届政府的开局之年。我们将全面做好稳增长、促改革、调结构、惠民生、防风险等工作，促进经济平稳健康发展、社会和谐稳定，以优异成绩迎接党的十九大和省第十一次党代会胜利召开。

今年发展的主要预期目标是：地区生产总值增长8%左右，地方一般公共预算收入增长8%，全社会固定资产投资增长10%左右，社会消费品零售总额增长10%，城乡常住居民收入增长与经济增长同步。全面完成省下达的节能减排任务。

重点抓好八个方面工作：

（一）加快产业转型升级

推进工业量质提升。开展“中国制造2025”城市试点示范。突出抓好100项重大工业项目。开工建设国家航天产业基地，支持华星光电T4、中兴通讯高能电池二期等一批重大项目开工；加快推进国家存储器基地、东风本田三厂、格力智能装备产业园等一批重大项目；确保武汉天马六代线、上汽通用武汉基地二期、华为武汉研发生产基地等一批项目投产。抓好乙烯扩能等20个重点技改项目建设。工业投资增长12%。开展智能制造推广行动，建设国家智能制造产业基地。创建全国“质量强市”示范城市。培育更多大城工匠。规模以上工业增加值增长8%。

推进服务业能级提升。引进区域金融总部5家，新增地方法人金融机构3家以上。引导大型商业综合体向社交体验、时尚消费、功能多元转变；培育壮大本土细分领域电商品牌，电子商务交易额增长25%；支持汉口北开展国家市场采购贸易方式试点。加快武汉中央商务区、武汉国际博览会展区、红钢城商务城建设，启动建设杨春湖商务区。

推进新经济加快发展。发展工业互联网、物联网、网络视听、远程诊断维护等新业态新模式。加快建设国家网络安全人才与创新基地。在人工智能、智能穿戴、虚拟现实、量子通信、工业机器人、大数据、无人机等新兴领域，培育“从0到1”的标杆企业、“从1到N”的领军企业。战略性新兴产业产值增长15%。

（二）加快提升自主创新能力

系统推进全面创新改革试验。突出抓好25项国家授权先行先试事项落地见效。发挥东湖国家自主创新示范区创新引擎作用。深化科技金融改革。积极创建国家知识产权强市。

强化企业技术创新主体地位。围绕信息技术、生命健康、智能制造等三大领域17个细分行业延伸创新链条，抓紧创新型企业培育引进、创新平台建设、关键技术与创新产品开发。新建企业研发中心100家，引进世界500强、中国500强企业研发机构30家。探索建立企业主导科技项目立项等激励机制。设立促进科技成果转化专项基金，推动创新成果产业化。高新技术企业新增300家。

激发双创活力。加快“创谷”建设，新建实体空间300万平方米，新引进科技企业和创业团队300个，打造4个国家级创新平台和双创基地，新增国家级众创空间20个。推进大学之城示范区建设，面向大学生开展校地、校企联合培养，培训青年创新创业人员2万名。创新人才发展体制机制，引进150名“城市合伙人”。

（三）加快提升城市功能品质

规划建设亮点区块。加快编制新一轮城市总体规划、土地利用总体规划、综合交通规划。建立高规格领导机构、高效率协调机制，高水平推进亮点区块建设。科学确定长江新城、长江主轴的范围，面向国际招标，编制总体规划、控制性详细规划及专项规划。集成优化长江主轴城市设计，实施一批牵引性项目，加快建设服务业聚集区，加快建设江北快速路，开工汉正街隧道、临江大道综合管廊，启动“梦幻江城”灯光秀二期，建设琴台美术馆，规划打造城市阳台。推进东湖城市生态绿心建设，续建东湖绿道60公里，全面提升东湖景观水平；加强大东湖地区环境和交通整治，继续实施截污工程。

强化综合交通枢纽功能。全面完成天河机场三期工程。发展多式联运，建成阳逻港集装箱铁水联运工程，推进江北铁路二期、新港高速等项目。争取新一轮《武汉铁路枢纽总图规划》尽快获批。推动武汉至杭州高铁尽早开工。抓紧建设光谷火车站。建成武深高速武汉段、硚孝高速武汉段。

完善快速交通体系。战略性提速轨道交通建设，加快推进11条在建线路，建成8号线一期、21号线，开工6号线二期、8号线三期、12号线，争取开工16号线、前川线。建成沌口长江大桥，开工建设江汉七桥，加快扩建月湖桥、岱黄高速府河桥，抓紧推进四环线、外环东扩、汉江大道等项目。

制定实施“四水共治”行动计划。防洪水，加固举水西堤郑园段、府河西湖堤、新合堤、童家湖拦渍堤等堤防，完成邾城防洪圈建设，整治病险水库35座。排涝水，新建排水干网100公里，建成江南泵站、南湖出江通道、夹套河骨干箱涵、黄浦路泵站、港西二期泵站等12个重点排水项目，推进北湖地区排水设施建设，中心城区抽排能力提高50%，完成青山、汉阳“海绵城市”示范区建设。治污水，推进18条河流黑臭水体整治，完成三金潭、黄家湖污水处理厂改扩建，启动北湖污水处理厂建设，改扩建南太子湖、汉西污水处理厂。保供水，新、改、扩建供水管道50公里，完成军山水厂建设、沌口水厂扩建、余氏墩水厂升级工程。我们要把“四水共治”作为生命线、发展线工程，下决心、大气力、高投入，兴水利、除水患、解水忧，让水清水灵水韵，润泽江城，造福市民！

加大绿化建设和土壤治理力度。实施快速通道绿化200公里，建成四环线生态林带23公里。中心城区新增花卉面积10万平方米。造林绿化2.5万亩。制定实施土壤污染防治工作方案。从严从实抓好中央环保督察突出问题整改。

推进城市管理升级。出台城管执法体制改革意见。提升环卫设施、空间立面、清扫作业、桥隧养护等标准。建成长山口生活垃圾预处理场，加快千子山循环经济产业园建设，提高垃圾减量化、资源化、无害化处理能力。深入实施违法建筑、户外广告、城中村和城乡接合部等专项整治。

（四）加快推进改革攻坚

深化供给侧结构性改革。坚持去产能与优产能相结合，在搬迁改造与转型升级中去落后产能，提高工业有效供给能力。促进房地产市场平稳健康发展，住房新开工面积、竣工面积均增长10%左右。积极稳妥降低企业杠杆率，直接融资增长15%。加大减税、降费、降低要素成本工作力度，力争为企业减负200亿元以上。

加大“放管服”改革力度。完善区级行政审批3.0改革，启动市级行政审批3.0改革，推动行政审批4.0改革。强化公共资源交易综合监管。加强事中事后综合监管平台建设。实现“双随机一公开”监管全覆盖。整治规范与行政审批相关的中介服务，引入充分竞争机制。

推进国企民企共同发展。深化混合所有制改革,积极开展国有企业股权激励、市场化选聘职业经理人试点。完善落实加快民营经济发展的政策措施。积极发挥商会、行业协会等作用，举办系列民间资本投资大会。加快推进10个全国PPP试点项目落地实施。

（五）加快发展开放型经济

扎实推进自贸试验区建设。制定湖北自贸试验区武汉片区实施方案，落实第一批20项重点试验任务。大力推进投资贸易便利化，探索实行市场准入负面清单制度，建立“一线放开、二线安全高效管住”的通关服务和检验检疫模式。

掀起招商引资热潮。强化考评考核，强化项目库建设，创新招商方式。对来汉投资贡献突出者，给予尊荣和礼遇。面向全球聘请招商大使，重奖招商功臣。设立招商引资投诉处理中心，对损害投资软环境的行为零容忍。各开发区和新城区至少引进1个投资50亿元以上工业项目，各中心城区至少引进1个投资30亿元以上服务业项目。确保招商引资总额增长16%以上，实际利用外资增长13%以上。我们要创新政策，强化激励，让广大干部打消顾虑、放手招商，让广大客商打消顾虑、放心投资，让更多大项目好项目落户武汉！

扩大对外贸易和投资。全面开展国家服务贸易创新发展试点，争创国家跨境电商综合试验区。加强国际产能合作，支持在“一带一路”沿线建立海外产业基地，带动装备与工程服务走出去。

加强大通道大平台大通关建设。新开通国际及地区航线3条以上，发展国际全货机航线。确保武汉新港空港综合保税区和阳逻港进口肉类指定口岸封关运行，加快武汉多式联运海关监管中心建设。积极申报汽车整车进口口岸，大力推进武汉电子口岸·国际贸易“单一窗口”建设。推动俄罗斯设立驻汉总领事馆。理顺管理体制，加快中法生态示范城建设。办好长江中游城市群省会城市第五届会商会。

（六）加快农业农村发展

高质高效发展现代都市农业。推进农业供给侧结构性改革，发展有机生态循环农业，支持发展“互联网+品牌农业”。深化农村土地“三权分置”改革，扎实推进农村承包土地的经营权、农民住房财产权抵押贷款试点，增强农村产权交易市场功能。加快建设7大现代都市农业示范园区。培育发展家庭农场、专业大户、农民合作社、农业龙头企业，新型农业经营主体达1.7万个。推广应用新品种、新技术、新模式100项以上。加快“中国种都”建设。乡村休闲旅游收入达86亿元。

努力建设美丽乡村。扎实推进新城区美丽乡村带和一批特色小镇建设。加强农村公路养护管理。切实解决老区人民的饮水、交通等生产生活问题。强化农业面源污染治理，严控“三区”、拆除“三网”，加大农村畜禽水产养殖污染治理力度。完善农村垃圾收运处理体系，确保垃圾无害化处理率90%以上。

（七）加强保障改善民生

织密扎牢就业社保安全网。推进创业富民，城镇新增就业18万人，扶持创业3万人，带动就业10万人。全面启动统一的城乡居民医保制度，社保扩面新增30万人次。城乡低保综合提标10%以上。实施棚户区改造6.85万户，建成保障房4.5万套。实现D级危房全面消危。

发展更高质量更加公平的教育。出台实施新一轮学前教育三年行动计划，推进义务教育优质均衡发展等改革，扩大优质教育资源覆盖面，缓解新建小区居民子女就近入园入学难。加快发展现代职业教育。提升城乡教育一体化水平。

全方位全周期保障市民健康。全面深化公立医院综合改革，降低大型医用设备检查治疗价格，严控不合理检查和化验费用，切实减轻群众负担、提升服务质量。积极构建医联体、健联体。培育名医、打造名科、建设名院。大力发展高端精准医疗，支持筹建质子重离子治疗中心及治疗设备研发制造，支持建设国际医院。大力发展全民健身。

提升城市文化品位。推进各类公共文化设施和文化功能区提档升级。推进归元古城文化区建设。加快盘龙城国家考古遗址公园建设，筹建武汉图书馆新馆、武汉自然历史博物馆。抓紧筹备第七届世界军人运动会，办好武汉国际马拉松赛、世界飞行者大会等品牌赛事。

加强和创新社会治理。在街道（乡镇）政务服务中心整合设立综合服务窗口，加快实现“一窗多能、全科服务、全区通办”。深化“三社联动”，提升基层社会治理能力。推动各类公共数据集成共享。实施阳光信访进社区（村）工程，覆盖率达100%。争创国家食品安全城市。加强安全隐患排查，强化建设工地、危化品等重点领域安全整治，坚决防范重特大事故发生。全面实施“七五”普法。深入开展双拥共建活动，加大国防教育力度，大力支持国防建设和军队改革，推动军民融合深度发展。

今年，我们将继续办好十件实事。

1、缓解交通拥堵。深化智慧交通系统建设，实现行车停车双诱导。建成微循环道路100条。依法整治100处交通堵点。新增公共停车位1.5万个、人防地下停车位1.2万个。

2、改善空气质量。加强工业大气污染防治，完成20万千瓦及以上燃煤锅炉火电机组超低排放改造，强化施工工地、道路扬尘管控，落实监管责任和智慧管理措施。空气质量优良率达到67%以上。

3、深化“互联网+政务服务”。建设全市统一的身份认证体系、政务服务标准规范、网上政务服务大厅、电子证照库。新增47项市级行政审批和公共服务事项网上全程办理。

4、精准扶贫济困。减少贫困人口47528人、贫困村107个。完成农村5.8万座户厕改造。为本市户籍18周岁以下患白血病、重型再生障碍性贫血等重大疾病的特困患者，一次性提供5万元以内的人道救助。

5、改善养老服务。城乡居民基本养老保险基础养老金增加20%。完善信息管理系统，实现老年证和高龄津贴网上帮办、服务到人。推广“互联网+居家养老服务”新模式。创建200个“老年宜居社区”。

6、提升教育医疗水平。实行市、区属中等职业教育免除学费。新、改、扩建公益普惠性幼儿园30所，新增幼儿入园学位4800个。新、改、扩建20所中小学校，新增学位1.8万个。建设青少年寒暑假社区托管室200家。推进市、区属公立医院就诊“一卡通”、病历“一本通”、同级医院检验化验结果“一单通”。

7、提升智慧应用水平。建设电梯安全监管智慧系统、排涝泵站智慧管理系统、25座重点桥隧安全监管智慧系统。推进城市视频监控系统三期建设。在重点公共区域建设公益免费无线局域网热点195个。

8、提升安全保障水平。在全市普通公路1000处急弯陡坡、临水临崖等危险路段设置安全防护设施。在人员密集重点场所建立流动警务服务站50个。规范建设100家农贸市场蔬菜农药残留快速检测室。开展食品安全抽样检测3万批次，每月公布抽检结果。

9、改善市容环境。建设绿道270公里、林荫道80条、街心公园40个、郊野公园5个。创建市容环境示范路16条。新、改建150座生活垃圾收集站（屋）。

10、推进文体惠民。为510支基层群众文艺团队配置活动器材。开展“家庭教育公益大讲堂”进社区（村）2000场。在40家游泳场所为中小学生提供20天免费暑期游泳。对6000名青少年开展游泳、足球、网球、篮球、羽毛球免费培训。

（八）加强政府自身建设

“政者，口言之，身必行之。”我们坚决不当“口号政府”，坚持做“行动政府”，努力建设人民满意政府。

践行忠诚，从严治政。牢固树立“四个意识”，特别是核心意识、看齐意识，始终在思想上政治上行动上同以习近平同志为核心的党中央保持高度一致。严守政治纪律和政治规矩，坚决贯彻党中央国务院、省委省政府和市委各项决策部署。认真落实全面从严治党主体责任和“一岗双责”，深入推进政府系统党风廉政建设和反腐败斗争，建设廉洁政府。

尊崇法治，依法行政。切实将政府活动全面纳入法治轨道。完善政府规章制定程序。建立执法全过程记录制度。主动接受人大法律监督、工作监督和政协民主监督，强化监察、审计监督，畅通社会监督渠道，让权力公开透明、阳光运行。

创新治理，高效善政。运用互联网思维方式和工作方法，以服务群众为根本、城市治理为己任、智慧应用为抓手，提高数字治理能力。强化大数据、云计算政务运用，提升决策管理水平。走好网上群众路线，主动从网上发现问题、吸收意见、推进解决，并形成常态机制。充分利用“三微一端”新平台，为市民提供多样化、个性化的定制服务和推送服务。

改进作风，务实施政。以时不我待、舍我其谁的担当精神，干事创业，奋发有为。务实重行，不浮躁、不浮夸、不漂浮，当“工匠”“钉钉子”，抓落实、求实效，全面推行政府工作项目化，有项目就有责任人、时间表、路线图，在落细落小落实中谋大事、干大事、成大事。加强履职能力和作风建设，切实提升行政效能。落实中央八项规定，深入纠正“四风”，从群众急切盼望的事情抓起，从社会反映集中的问题改起，全面整治庸政懒政怠政，用群众切身感受评判工作效果，努力让群众对政府工作过程和结果双满意。

各位代表！乘风好破浪，奋进正当时。让我们更加紧密地团结在以习近平同志为核心的党中央周围，在省委省政府和市委的坚强领导下，拼搏赶超，奋勇争先，把建设现代化、国际化、生态化大武汉的时代主题，书写在武汉发展的旗帜上，让复兴大武汉的时代凯歌，响彻在江城希望的大地上，共同创造更加幸福美好的明天！
